# Complex Degradation Processes Lead to Non-Exponential Decay Patterns and Age-Dependent Decay Rates of Messenger RNA

**DOI:** 10.1371/journal.pone.0055442

**Published:** 2013-02-11

**Authors:** Carlus Deneke, Reinhard Lipowsky, Angelo Valleriani

**Affiliations:** Department of Theory and Bio-Systems, Max Planck Institute of Colloids and Interfaces, Potsdam, Germany; University of California San Diego, United States of America

## Abstract

Experimental studies on mRNA stability have established several, qualitatively distinct decay patterns for the amount of mRNA within the living cell. Furthermore, a variety of different and complex biochemical pathways for mRNA degradation have been identified. The central aim of this paper is to bring together both the experimental evidence about the decay patterns and the biochemical knowledge about the multi-step nature of mRNA degradation in a coherent mathematical theory. We first introduce a mathematical relationship between the mRNA decay pattern and the lifetime distribution of individual mRNA molecules. This relationship reveals that the mRNA decay patterns at steady state expression level must obey a general convexity condition, which applies to any degradation mechanism. Next, we develop a theory, formulated as a Markov chain model, that recapitulates some aspects of the multi-step nature of mRNA degradation. We apply our theory to experimental data for yeast and explicitly derive the lifetime distribution of the corresponding mRNAs. Thereby, we show how to extract single-molecule properties of an mRNA, such as the age-dependent decay rate and the residual lifetime. Finally, we analyze the decay patterns of the whole translatome of yeast cells and show that yeast mRNAs can be grouped into three broad classes that exhibit three distinct decay patterns. This paper provides both a method to accurately analyze non-exponential mRNA decay patterns and a tool to validate different models of degradation using decay data.

## Introduction

In each cell, the information encoded in the DNA is transcribed into messenger RNAs (mRNAs), which then, in turn, are translated into proteins. From a single-molecule point of view, each mRNA has a finite lifetime and its decay arises from the action of a variety of degrading enzymes that break down the mRNA into its constituents, the nucleotides. The encounters between mRNA and degrading proteins are largely dominated by stochastic effects.

Given the relevance of mRNA concentration on protein abundance [1]–[3], much effort has been dedicated to improve our understanding of mRNA degradation [4]. In the past decades, a number of different mechanisms responsible for the degradation of the mRNA have been identified [5]–[9]. Some mechanisms of degradation are known to affect the decay of all mRNA species and are thus unspecific. In contrast, other mechanisms are known to affect certain mRNAs more than others depending on different physical and chemical properties of the nucleotide chain. For example, micro-RNAs mediate the docking of degrading enzymes specifically to each mRNA and contribute thus to the large variation of the stability between mRNA species [8]–[13].

One widely studied degradation pathway in bacteria is known as *endonucleolytic* degradation [5]. This degradation process is initiated by cleavage within the nucleotide chain by the action of a single protein or protein complex. For instance in *E. coli*, RNAse E and its homologues are proteins responsible to initiate endonucleolytic decay. Once the degradation process has been initiated, it leads to a rapid decay of the attacked mRNA with a sudden interruption of translation. In this case, the time scale related to the random encounter between the degradation complex and the mRNA primarily determines the lifetime of the mRNAs. It is commonly believed that eukaryotic mRNAs are affected to a lesser extent by endonucleolytic degradation than prokaryotic mRNAs [5]. In eukaryotic cells, the most common mechanisms of degradation are those that lead to *decapping*. This mechanism requires deadenylation at the 3′ region and the destabilization of the 5′ cap structure before degradation occurs in the 5′ to 3′ direction behind the last translating ribosome [5], [14]. Different exonucleolytic degradation pathways exist also in bacteria. In *E. coli*, for instance, modification of the 3′ stem-loop is a prerequisite of exonucleolytic degradation initiation [5]. Moreover, in *B. subtilis* a 5′ exonuclease has been discovered recently [15], [16]. Furthermore, a variety of miRNA and small-RNA mediated degradation mechanisms have been identified [8], [9], [11], [12]. These mechanisms require several biochemical steps for complete degradation or complete loss of functionality.

Irrespective of the degradation pathway, the lifetime of a single mRNA is a random variable that will depend on the diffusion time of the degrading complexes and on the time scale of enzymatic activity at the various steps of degradation. Moreover, the particular form of the lifetime distribution for each species of mRNA depends on the specific mechanisms that are responsible for its degradation. A species of mRNA that is mostly degraded by the action of an endonuclease, for instance, will have an exponential lifetime distribution. The same holds also for degradation processes which involve only one relatively slow, rate-limiting step.

In contrast, during the decapping process of degradation or during the degradation process triggered by miRNA, the mRNAs undergo a series of biochemical modifications [11], several of which are characterized by relatively slow rates, which implies that their lifetime distribution cannot be described by a single exponential function.

This simple observation has dramatic consequences. Indeed, a basic result in probability theory states that the exponential distribution is memoryless, *i.e.* the life expectancy does not depend on the age of the process, while any other probability distribution carries a memory of the past. For the mRNAs, this memory is encoded in the biochemical transformations or in other transient phenomena that characterize the aging of the mRNA.

In this paper, we show that complex mRNA degradation processes necessarily lead to lifetime distributions that are not exponential. Our study addresses the relationship between the mRNA lifetime distribution and the experimentally observed mRNA decay patterns. The diverse degradation processes described above call for a general theory of mRNA degradation. The theory that we present here provides a robust mathematical framework, into which one can incorporate additional molecular details about specific degradation mechanisms.

Experimentally, the decay patterns are often determined by the measurement of the decaying average amount of each mRNA species at steady state expression, in a cell culture after the interruption of transcription. This method proceeds by taking several samples at different time points, as described in [17]–[23]. The data points are then fit by an exponential function in order to compute the half-life of the mRNAs. However, this fitting procedure has many shortcomings. In Ref. [17] a considerable amount of data have been eliminated because they could not be fit by an exponential function and in Refs. [18], [19] many rather distinct decay patterns were observed so that the idea of a fit with a simple exponential has been rejected for the majority of the mRNAs.

**Figure 1:**
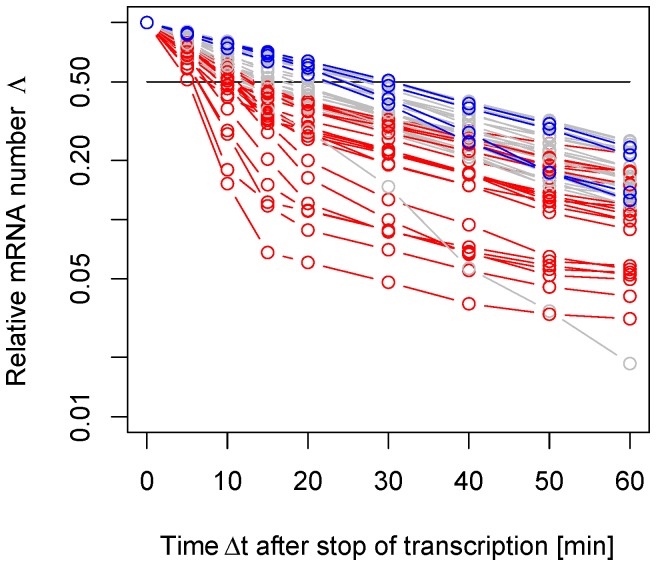
Experimental mRNA decay patterns. The relative mRNA number 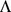 defined in Eq. (1) can be measured at different time points after the interruption of transcription for *S. cerevisiae* as adapted from [23]. In this semi-log plot we show only those decay patterns that are monotonically decreasing and satisfy the convexity properties according to the general condition derived in Eq. (18). From the 51 decay patterns shown here, 21 curves show a cross-over from fast to slow decay (red) while 4 curves show a cross-over from slow to fast decay (blue). This indicates that the purely exponential decay is only one of several possible decay patterns.

Fig. 1 reproduces some of the measured decay curves for *S. cerevisiae* from Ref. [23]. We highlighted those patterns that strongly deviate from an exponential decay. The red patterns show a marked cross-over from a quick decay at short time scales to a slow decay at larger time scales. The blue patterns, instead, show the opposite behavior with a cross-over from a slower decay at short time scales to a quicker decay at large time scales. In the background, the gray lines show those decay patterns that are approximately exponential. In particular, the analysis of the data shows that short-lived mRNA species tend to belong to the set of the red decay patterns while long-lived mRNAs tend to belong to the set of the blue patterns in Fig. 1.

The inhibition of transcription is likely to strongly stress the cells and possibly leads to undesired side effects. Therefore, several laboratories have developed an alternative method and applied the non-perturbing pulse-chase technique to assess mRNA stability [24]–[26]. This method is based on the labeling of the mRNAs with a heavy nucleotide, which is added to the cell culture over a period of time. Again, a measurement of the relative amount of labeled mRNAs over time reveals the time scale of degradation for the observed mRNA species. However, also here the pattern may or may not be exponential and the quality of the data relies on the number of recorded time points and on a good choice of the fitting curve, which reflects the assumptions about the biochemical mechanisms for the degradation process.

In this article, we will first derive a general relationship between the mRNA lifetime distribution and the decay patterns for the amount of mRNA, starting from a steady state expression level. Furthermore, we will introduce the concept of mRNA aging and show that the residual lifetime distribution of the mRNAs as well as their potential protein yield change during the experimental determination of the half-life. Finally, we will develop a model for degradation by multiple steps and apply it to data of *S. cerevisiae* in order to gain insight into the form of the associated lifetime distributions and a possible classification of the decay patterns of the entire mRNA pool.

## Results and Discussion

**Figure 2:**
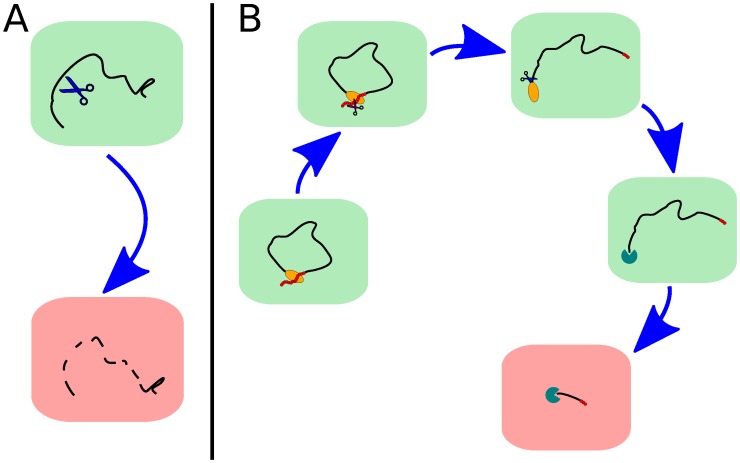
Prototypical pathways of mRNA degradation. In panel A degradation is depicted as a relatively simple process determined by only a single step, e.g. by unspecific and fast endonucleolytic decay, such as the degradation pathway mediated by RNase E in prokaryotic cells. In panel B, instead, we show a schematic representation of the degradation pathway known as *decapping* which is one of the main degradation mechanisms in eukaryotic cells. The decapping mechanism consists of several biochemical steps, possibly triggered by a specific miRNA, which contribute to destabilize the mRNA until complete degradation takes place. This mechanism can be considered as a prototype of multi-step degradation.

First, it is important to distinguish between single-step and multi-step degradation. On the one hand, endonucleolytic degradation, depicted in Fig. 2A, is a prototype of single-step degradation. On the other hand, the decapping mechanism shown in Fig. 2B is a prototype of multi-step degradation. The lifetime distribution of the mRNAs will resemble an exponential function if there is only one rate limiting process relevant for degradation as in Fig. 2A. Conversely, the lifetime distribution will have a more complicated form if several biochemical modifications are necessary as in Fig. 2B, and it will be directly related to the details of the particular degradation pathway. Relatively simple processes like those illustrated in Figs. 2A and 2B can be described within the framework of continuous-time Markov chains, which is a common mathematical tool in stochastic modeling of biological processes (see *Models and Methods* for details). More complex degradation processes may instead require different models. Nevertheless, there exists a general mathematical framework that links the degradation process to the decay pattern.

### From Lifetime Distributions to Decay Patterns of mRNA

From a single-molecule point of view, the degradation process determines the lifetime of each individual mRNA molecule. Since the degradation process is largely dominated by random events, it is appropriate to consider the lifetime of an mRNA as a random variable. Thus, the most important quantity in our theory is the probability density 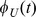 for the random lifetime 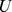 of an mRNA molecule. The density 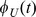 can be determined either empirically from data (see below) or theoretically, if all details of the degradation process are known.

Once the function 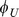 has been determined, one can compute the analytical form of the decay pattern for the amount of mRNA after the interruption of transcription. Our theoretical framework accounts for the stochasticity of transcription and the random lifetime of the mRNAs. These random effects lead to fluctuations in the mRNA amount that are intrinsic to mRNA turnover (see section *Models and methods*). Nevertheless, to explain the experiments on mRNA decay, one just needs to describe the time evolution of the average number of mRNA.

We first consider the situation, in which the population of each mRNA species is at steady state. Under this condition, let 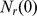 denote the average number of one species of mRNA at the time at which transcription has been interrupted, and let 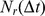 denote the amount of this mRNA species at time 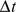 after the stop of transcription. The relative number of mRNAs per cell as defined by 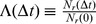 is then given by
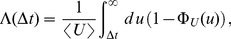
(1)where 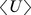 is the average lifetime of an mRNA molecule and 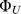 is the cumulative lifetime probability defined as
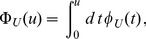
(2)which describes the probability that an mRNA is degraded before time 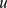. Thus, the decay pattern of 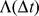 depends therefore on the form of the lifetime probability density 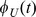, which is determined by the biochemical processes that lead to the degradation of the mRNA. As mentioned, the computation leading to Eq. (1) assumes steady state, in which mRNAs of all ages are present in the sample. From the experimental point of view, it is sometimes convenient to study a cohort of mRNAs that have been synthesized in a pulse of transcription. In this case, all mRNAs have approximately the same age and Eq. (1) is replaced by



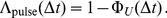
(3)In the remainder of this work, we will focus on the analysis that assumes a steady state expression level of the mRNA and consider Eq. (1) as the function that gives the decay pattern of the mRNAs.

A further consequence of Eq. (1) is that the shape of the decay pattern must fulfill a certain convexity property as will be explicitly shown in Eq. (18). Thus, only those patterns that decrease monotonically with a non-negative second derivative are consistent with a meaningful (*i.e.* positive) lifetime probability density. This criterion provides a general constraint on the decay pattern without assuming any specific degradation mechanism. All decay patterns shown in Fig. 1 obey this *bona-fide* criterion, however many other patterns deduced from the same experiment do not, which presumably reflects the perturbing nature of experiments that block transcription globally. We must notice, however, that the convexity constraint does not hold if the cohort of mRNA originates from a short transcriptional pulse.

### A Stochastic Theory of Multi-step mRNA Degradation

The lifetime distribution 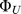 of each mRNA species reflects the characteristics of the degradation process of that species. By inverting Eq. (1) one could in principle determine the lifetime distribution 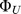 directly from the experimentally observable decay pattern. However, to obtain accurate results it would be necessary to have a high temporal resolution in the experimental determination of 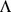. Therefore, it is useful to construct a model that reflects both some characteristics of the underlying degradation mechanisms and allows a good fitting of the decay data even at a relatively low temporal resolution. The inspiration for such a model comes from the biochemistry of mRNA degradation. In Fig. 2 we have illustrated two prototypes of mRNA degradation, classified according to being a single or a multi-step degradation process. A general and useful tool to model multi-step processes are Markov chains. Here, any biochemical state is mapped onto a state of the Markov chain and a biochemical modification of the mRNA is reflected by a transition between states of the chain (see *Models and Methods*). Such a model determines the functional form of 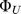 and is flexible enough to describe the different decay patterns of the various mRNA species in the cell. Furthermore, this approach opens the possibility to mathematically study different biochemical models and to check their validity by comparing their predictions to experimental data via Eq. (1).

**Figure 3:**
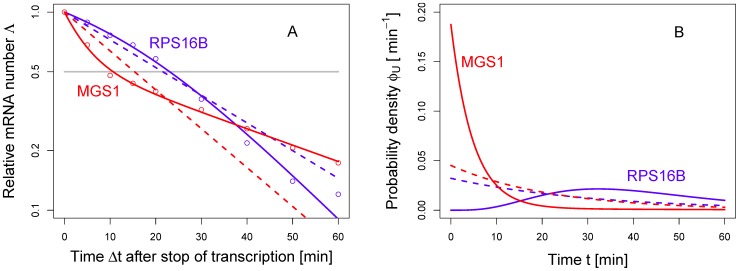
mRNA decay patterns and lifetime distributions. Relative mRNA number 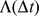 as a function of the time 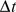 after the interruption of transcription. (A) Two different experimental decay patterns are reproduced from [23] (circles) corresponding to the genes *MGS1* (red) and *RPS16B* (blue) of *S. cerevisiae*. The solid lines represent decay patterns as calculated by the Markov chain model and the corresponding rates are estimated from a non-linear regression analysis (see *Models and Methods* for details about the fit parameters). For comparison, we also show a fit with a simple exponential function (dashed lines) which is clearly not suitable to capture the entire information of the degradation process. In particular, the exponential fit for the red data points suggests a half-life (intersection with the horizontal line) that is almost twice as large as the true half-life. (B) The corresponding lifetime densities 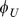 are derived using the rates obtained via the fit of Eq. (1) with the data. Evidently, both densities differ strongly from an exponential distribution indicated by the dashed lines. While the red line shows a rapidly decaying lifetime distribution, the blue line is broadly distributed, with a clear maximum at an intermediate time.

By exploiting our Markov model, in Fig. 3A we analyzed two instructive examples from the experimental decay patterns in Fig. 1: The mRNA encoding *MGS1* (Maintenance of Genome Stability 1, red) and the small ribosome subunit protein (*RPS16B*, blue). A comparison between the best fit with an exponential function and the best fit with *a multi-step* model shows that the latter is clearly more appropriate than a single exponential in describing the decay profiles over the entire time course of the experiment. Furthermore, in the case of *MGS1* mRNA, the fit reveals a half-life 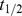 which is substantially smaller than the estimated half-life from an exponential fit. We can thus observe that a measurement of the half-life 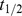 is not, in general, a good measure of the average lifetime 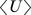 of the mRNAs and it fails to predict the decay rate accurately.

### Age-dependent Rates of mRNA Degradation

Fig. 3A reveals that for the gene *MGS1*, degradation of the corresponding mRNA becomes less efficient during the lifetime of the molecule. In contrast, for the gene *RPS16B*, mRNA degradation becomes effective only after a transient time. In both cases, we concluded that the effective degradation rate 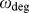 depends on the age 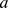 of the mRNA. In fact, the age-dependent degradation rate 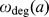 can be expressed in terms of the lifetime probability density 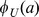 and the cumulative lifetime probability 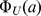, according to the simple relation
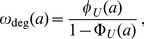
(4)which is often termed the hazard or failure rate in the literature [27] and is frequently used to describe the age-dependent division process in cell populations [28], [29]. The rate 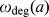 determines the probability of degradation of an mRNA of age 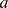 in the (infinitesimal) interval 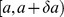. A heuristic derivation of Eq. (4) is given in the section *Models and Methods*.

**Figure 4:**
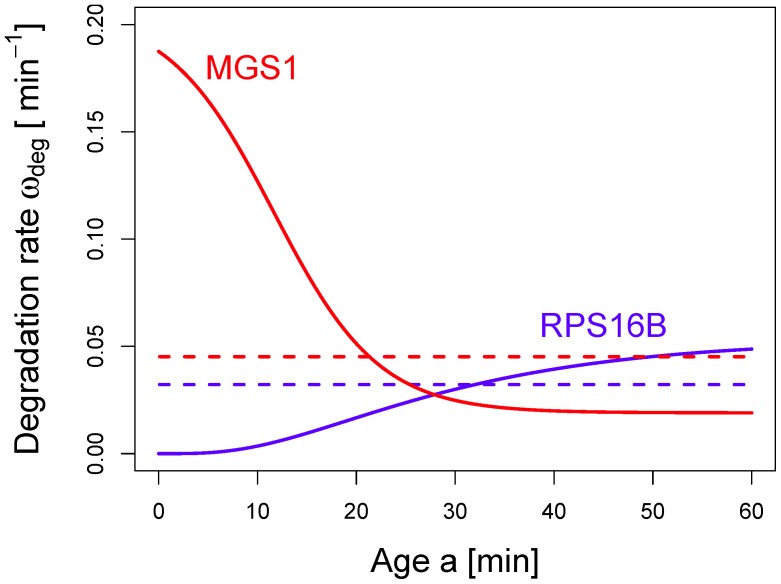
Effective degradation rate 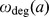 as a function of the age *a* of an mRNA. The lifetime distribution of an mRNA can be translated into an age-dependent degradation rate 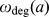 via Eq. (4). Here, we illustrate the change of the degradation rate during the lifetime of an mRNA for the two decay patterns shown in Fig. 3. For the mRNA encoding *MGS1* (red), the degradation rate is high for young mRNAs and decreases to a constant value after some transient time. In contrast, for *RPS16B* mRNA (blue), the degradation rate is close to zero upon birth of the mRNA and increases gradually to a constant value. For comparison, the constant rates corresponding to a fit of the decay data with purely exponential functions (dashed lines) are also included.

**Figure 5:**
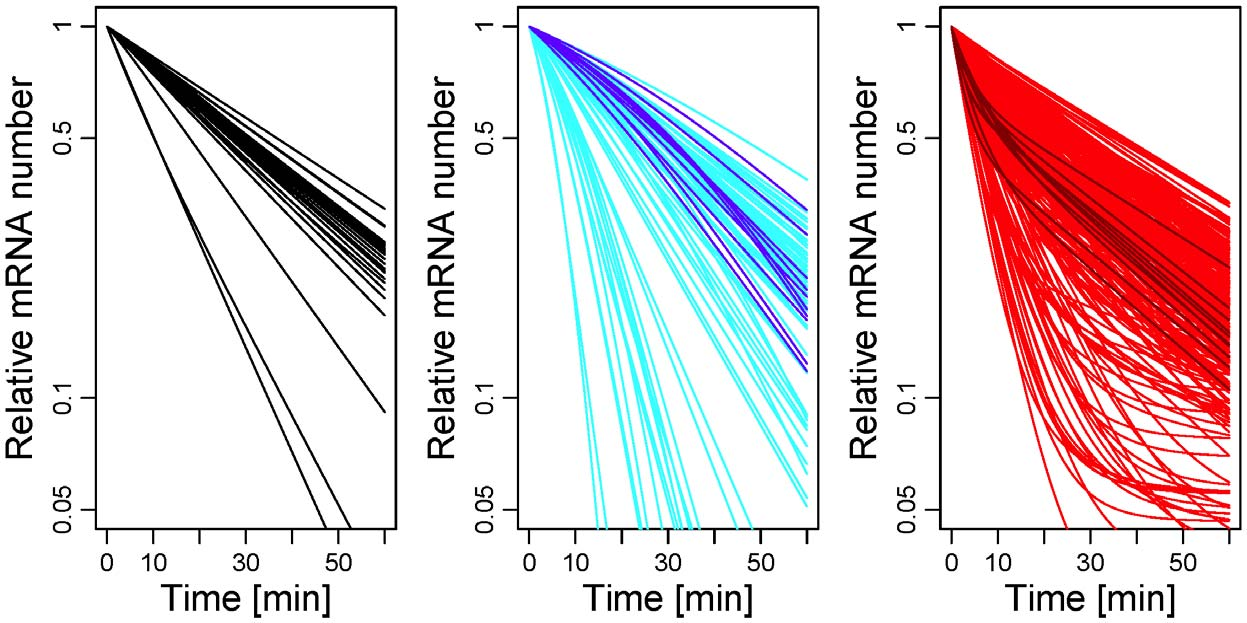
mRNA decay patterns in *S. cerevisiae*. The patterns are obtained from a systematic fitting procedure applying Eqs. (1) and (9) or (10) to the experimental data from Ref. [23]. The curves show the theoretical decay patterns that minimize the deviation between theory and experiment. Note that the experimental data points are omitted here for better legibility. The left panel shows 21 fitted curves that decay exponentially in good approximation (the best fit was either an exponential function or fitting by another function leads to an error reduction of less than 10 per cent). Conversely, 94 curves show a moderate decay followed by a fast decay (central panel, the best fit was obtained by Eq. (10) with 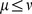) and 309 curves decay rapidly at short times and then level off (right panel, the best fit was obtained by Eq. (9) with 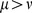). Thus, the majority of decay patterns does not follow a single-exponential decay law. For this visualization of the different categories, we considered data that were nearly *bona-fide* (see text) resulting in 424 genes. Moreover, in the central and right panel we highlighted a number of decay patterns that display a strong contrast to an exponential decay.

Fig. 4 illustrates the age-dependence of the degradation rates for the curves in Fig. 3. Clearly, the degradation rate varies strongly during the lifetime of the two chosen mRNAs. The two examples show that the changes of the degradation rates with age are qualitatively different for the two mRNAs. While *MGS1* mRNA is initially relatively unstable, the maturation of the molecules leads either to a stabilization of the mRNAs with their age or to a selection of stable mRNAs from the pool. This form of the age-dependent degradation rate indicates that strong degradation processes are at work before or during the relatively slow process of deadenylation. Phenomena such as differential nuclear mRNA degradation, mRNA storage in cytoplasmic stress granules and transient 3′ uridylation can in principle all lead to a reduction of the decay rate[30]–[32]. In contrast, young *RPS16B* mRNA is very stable but aging processes lead to its destabilization. This indicates that for *RPS16B* a series of relatively slow steps is necessary to complete the degradation process, in agreement with the picture provided by the decapping mechanism. A similar distinction is thus relevant also for the two non-exponential categories in Fig. 5 (see below).

Note that from the definition of 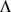 given in (1) one can derive the following relation
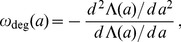
(5)by combining Eqs. (18) and the definition (4) of the age-dependent degradation rate. This relation shows that the age-dependent decay rate 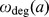 can be directly deduced from the measured decay pattern, when the latter has been determined with sufficient precision. Hence, the conclusions drawn from Fig. 4 could, in principle, be obtained without specification of a detailed decay model. If the experiment is performed as a transcriptional pulse, the relation between 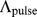 and 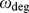 is given by
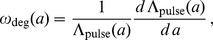
(6)where 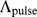 was defined in (3). Note that (5) and (6) provide the same age-dependent degradation rate from two different experimental procedures.

### Residual and Functional Lifetimes

**Figure 6:**
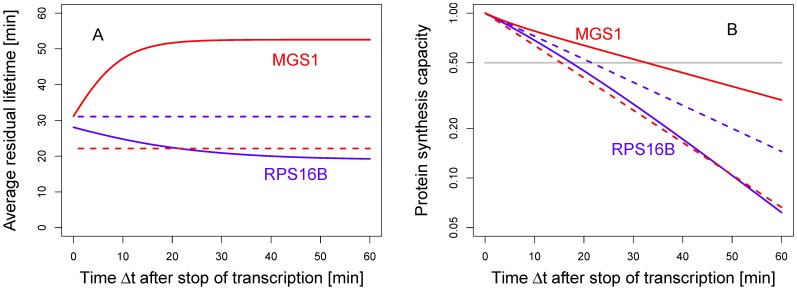
Average residual lifetime and residual protein synthesis capacity. (A) Average residual lifetime 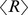 as function of time 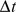, as defined in Eq. (20), after the interruption of transcription for *MGS1* (red) and *RPS16B* mRNA (blue). Under steady state conditions, *i.e.* 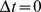, both have similar residual lifetimes. However, if transcription is stopped, the remaining mRNA population ages. The average residual lifetime of *MGS1* mRNA still present in the cell at time 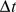 increases with 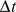 because only old mRNAs with a low degradation rate are still in the sample (see Fig. 4). In contrast, for *RPS16B* mRNA the average residual lifetime decreases. Only for exponentially distributed lifetimes (dashed lines) the average residual lifetime stays constant, which reflects the memoryless property of the exponential distribution; (B) Residual protein synthesis capacity 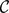 versus 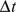 as defined in Eq. (7). The capacity 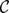 is proportional to the amount of proteins that will be produced by an average mRNA from the sample. The residual protein synthesis capacity decays exponentially if the mRNA has an exponential lifetime distribution (dashed lines) but follows a different pattern if the process of degradation is more complex. The small differences between the exponential and the true decay patterns indicate that the non-exponential character of the lifetime distributions may be difficult to deduce from measurements of the residual protein synthesis capacity 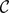.

It was shown that the aging of mRNA affects both the polysomal size distributions [33], [34] and the rate of protein synthesis [35]. From the point of view of mRNA degradation, aging becomes manifest in the residual lifetime *R* of the molecule. The residual lifetime of a randomly chosen mRNA is the remaining time until it is degraded. The average residual lifetime in a sample of mRNA molecules can be easily computed both at the beginning of the experiment, corresponding to the steady state, and during the decay assays (see *Models and methods*). Fig. 6A shows the behavior of the average residual lifetime 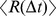 as a function of time 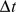 after the interruption of transcription for the two mRNAs discussed in Fig. 3. One can clearly see that the average residual lifetime changes with time reflecting the aging of the mRNA population after the stop of transcription, which is a consequence of the non-constant degradation rate in Fig. 4. Because the remaining mRNAs are older, their average degradation rate has changed and, thus, the average residual lifetime can increase (*MGS1*) or decrease (*RPS16B*). Only the exponential fit shows no aging and a constant residual lifetime (dashed lines).

The residual lifetime is connected to the *functional lifetime*, which is defined as the time over which the mRNA is available for translation by ribosomes. The functional lifetime of an mRNA is estimated from the measurement of the number of proteins that can be synthesized by the remaining mRNAs still present at any given time point after the interruption of transcription [36], [37]. This number is referred to as the *residual protein synthesis capacity*. In a first approximation, the residual protein synthesis capacity 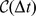 of an average mRNA is proportional to the number of remaining mRNAs 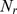 and their corresponding average residual lifetime 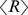 at any time 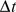 after the interruption of transcription. If we employ the normalization condition 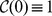, it can be defined as
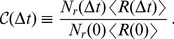
(7)


Hence, it is fully determined by Eqs. (1) and (20).

The plot of the residual protein synthesis capacity 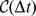 is shown in Fig. 6B. Note that only for exponentially distributed lifetimes the synthesis capacity follows an exponential decay since only in this case 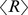 is a constant. Conversely, for more complex degradation processes, Fig. 6B indicates that the residual protein synthesis capacity 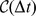 follows a different decay pattern for *MGS1* and *RPS16B* mRNA than the pattern predicted by the exponential fit.

### Global Analysis of mRNA Decay Patterns *S. cerevisiae*

The methodology developed so far allows us to analyze large sets of mRNA decay data and perform a classification of the mRNAs according to their decay pattern. In Fig. 5 we show the result of our systematic fitting procedure by using a (multi-step) Markov chain with 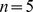 states. As mentioned earlier, the choice of the number of states is by no means unique and our analysis does not strongly depend on this choice. Our choice of 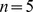 is inspired by biochemical studies that have identified five main steps in the process of mRNA degradation, see *e.g.* Ref. [11]. The classification method is based on a choice of parameters that minimize the residual sum of squares (RSS), for details see *Models and Methods*. For each decay pattern, we have attempted a fit with several variants of the multi-step model. The black decay patterns in Fig. 5 decay approximately exponentially and thus require just one parameter in order to obtain a fit that satisfies our criterion. The blue and the red curves in Fig. 5, instead, require at least three parameters in the Markov model in order to obtain a good fit (see *Models and Methods* for details). The most important conclusion from the decay patterns in Fig. 5 is that the majority of them do not follow an exponential decay. The blue curves in Fig. 5 exhibit a slower decay at short time scales and cross over to a faster decay for longer time scales.

One particular example is provided by the decay pattern of the mRNA *RPS16B* in Fig. 3. In these cases, the degradation rate increases with age of an mRNA, similar to the blue curve in Fig. 4. The red curves in Fig. 5 follow a different pattern: They decrease faster at shorter time scales and cross over to a slower decrease at larger time scales. This leads to an age-dependent degradation rate that is qualitatively similar to the one of *MSG1* in Fig. 4. One can notice, in Fig. 5, that some red and blue curves appear to decay close to exponentially. In the light of our results, one can consider the exponential decay pattern as a limiting case of the other two categories.

### Conclusions

Complex degradation pathways lead to lifetime distributions that cannot be described by a simple exponential distribution. This implies that the decay pattern is not exponential either. Such non-exponential patterns have been frequently observed, for instance in [19]. As a consequence, any attempt to fit these non-exponential patterns with a single exponential function leads, typically, to a bad fit and to the elimination of some data sets [17]. Whenever the degradation process can be described by a continuous-time Markov chain, all decay patterns tend to look exponential in the limit of long time scales. However, the time scale over which this exponential behavior becomes evident may be quite long and may vary strongly from one mRNA species to another.

Furthermore, our theory shows that by measuring only one time point in a decay assay - such as for instance in pulse-chase experiments - is, in general, not sufficient for a precise determination of the average lifetime of the mRNA. This kind of measurements, indeed, would deliver the correct decay rate only if such a rate does exist, namely only if the lifetime distribution were exponential. Detailed measurements of the decay pattern, such as those shown in Fig. 1, reveal however that an exponential decay represents just one of many possible decay patterns. Moreover, for a non-exponential lifetime distribution, the relationship between half-life and average lifetime is far more complex than for an exponential lifetime distribution. This implies that the half-life is in general not a good predictor of the average lifetime.

The counterintuitive relationship between half-life and average lifetime has important consequences for the comparison of mRNA decay patterns under two different growth conditions, say A and B. Because of the complex nature of the degradation pathway, it is, for example, possible that the half-life under condition A is greater than the half-life under condition B while, at the same time, the average lifetimes obey the opposite inequality. Therefore, it is, in general, not possible to compare the decay pattern of the same mRNA species under two different conditions by using only one single time scale. Furthermore, such a situation cannot be adequately described by the qualitative statement that one condition leads to a ‘faster decay’ compared to the other condition. Instead, a detailed analysis of the two decay patterns is now mandatory in order to draw any meaningful conclusions from the comparison of the two growth conditions. Indeed, only such an analysis can reveal those steps of the mRNA degradation process that are primarily affected by switching from condition A to condition B.

The theory presented here shows that there is a direct relationship between the biochemical details of a putative degradation pathway and the corresponding pattern in a decay assay. As such, our theory can be used to check if a putative decay mechanism is compatible with the observed decay pattern. The next theoretical and experimental challenge is to bring these two aspects of degradation closer together. Thus, one would like to use the experimental knowledge about the biochemical degradation pathway in order to predict the patterns measured during decay assays such as those shown in Fig. 1. At present, such detailed knowledge is still lacking and many quantitative details of the decay processes have still to be elucidated. Nevertheless, our theory provides a general, rather flexible framework for predictive models of mRNA turnover which can be refined by incorporating additional insights into the underlying biochemical processes. In this way, one should be able to identify the molecular mechanisms underlying the different functional forms for the mRNA lifetime distributions as deduced here from the observed mRNA decay patterns.

Typically, decay experiments are performed on non-synchronized cell populations where individual cells can be in different phases of the cell cycle. In general, the regulation of mRNA stability can change during the cell cycle. In a recent experiment, measurements of mRNA decay in yeast cells were confined to particular phases of the cell cycle [38]. There, it was found that for some mRNAs the decay patterns did not differ much in *S* and *M* phase, whereas others showed a distinct pattern. Moreover, the decay patterns observed during one particular phase were found to be non-exponential as well. In any case, to correctly assess changes in the mRNA decay behavior between different conditions or phases, it is necessary to formulate a theory of mRNA decay such as the one developed here.

Our theory allows us to suggest a new experimental procedure. Indeed, also the knowledge of the steady state distribution over the biochemical state space of each mRNA species would allow deriving its lifetime distribution. Therefore, experiments aimed at measuring the relative amount of mRNA in the different states could substitute experiments based on the stop of transcription and thus eliminate the undesired effects of this procedure. The theoretical foundation of this alternative procedure is a consequence of the theory presented here. However, the elucidation of all details goes beyond the scope of this study and will be developed elsewhere.

## Models and Methods

In this section we describe the models used to perform our theoretical investigation. We start by introducing a relatively detailed but rather intuitive Markov chain model, which maps the biochemical states of an mRNA during the degradation process as states of the chain. In the second part, we introduce the general mathematical theory of mRNA decay and aging, which is not based on any specific underlying model. This theory allows us to derive general results concerning the convexity properties of the decay patterns, the age dependent degradation rates, and the distribution of the residual lifetimes.

### Degradation Process as a Markov Chain

**Figure 7:**
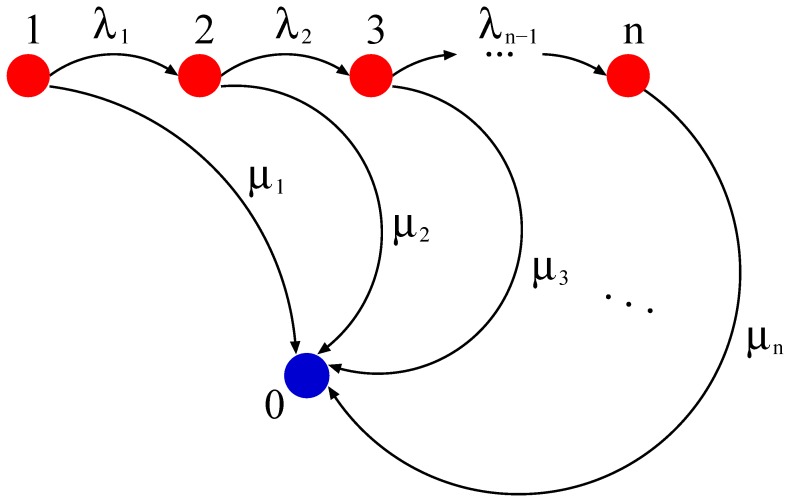
Maturation and degradation of mRNA viewed as a Markov chain. During its lifetime, each mRNA undergoes biochemical modifications described by transitions from state *i* to state 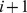 with a rate 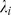. These alterations may result in a change of the degradation rate 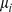 that governs the transition from state *i* to the absorbing state 0. The probability density of the time to absorption provides the distribution 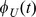 for the lifetime of the mRNA. Many known degradation pathways can be described in this manner and thus provide the mRNA lifetime distribution if quantitative information about the rates is available. This model can also be used to fit experimental data, as was done in Figure 3, in order to derive the empirical lifetime distribution.

From the prototypical degradation processes illustrated in Fig. 2 we develop a suitable mathematical framework in order to derive the associated lifetime distribution 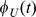. This can be done by translating the degradation pathways into the theoretical concept of a Markov chain described in Fig. 7. Here, any biochemical state is mapped onto a state of the Markov chain and a biochemical modification of the mRNA is reflected by a transition from one state to the next. In principle, many biochemical pathways of mRNA degradation can be described in this manner, provided one knows the details of the pathway. Markov chains, moreover, provide a well known tool in stochastic modeling with applications in many problems inspired by biology. One of the big advantages of Markov chains is the simplicity of their mathematical treatment. Some applications to single-molecule analysis similar to the one presented here can be found in Refs. [39]–[42].

In Fig. 7, the transition rates 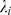 from one state *i* to state 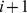 in Fig. 7 are governed by the time scale of the particular biochemical modification. For instance, the waiting time until arrival of the responsible enzyme or the time scale of its catalytic activity might be the rate limiting process for this step. The degradation corresponds in our model to a transition into the absorbing state. After every modification, *i.e.* in every state, degradation can in principle take place before reaching the next state of the decay chain. This degradation process is governed by the degradation rate 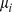, which can be, in principle, different for every *i*. From the theory of Markov chains we know that absorption, *i.e.* degradation of the mRNA, is certain but the associated time scale is random. In this mathematical framework, the lifetime density 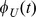 is the probability density of the absorption time.

For any given number *n* of modifications and for any choice of the rates, one can compute the lifetime probability density 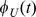 by solving the corresponding Master equation. However, the exact number of states related to each degradation process is not precisely known in most cases. It remains, therefore, an experimental and theoretical challenge to specify the relevant steps in the degradation process and their associated rates.

To obtain a qualitative understanding of the processes, we have applied some restrictions on the parameters. When all rates 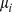 are identical, *i.e.* satisfy 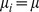, the lifetime probability density is completely independent of *n* and is given by
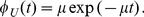
(8)


Note that this result holds independently of the choice of the rates 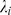. In Fig. 3A the dashed decay curves are derived from a model fit based on Eq. (8). The corresponding rates are 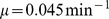 (red) and 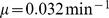 (blue), respectively. Once the rates have been determined, Eq. (8) yields a constant residual lifetime as in Fig. 6A and an exponentially decreasing residual protein synthesis capacity as in Fig. 6B. Also the black decay patterns in Fig. 5 are those for which no significant improvement of the fit was possible by taking a model more complex than Eq. (8). These decay patterns are thus genuinely exponential.

The best non-exponential fits in Fig. 3 (solid lines) have been obtained by using the Markov chain model in Fig. 7 with 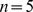 states and up to three independent rates, which were chosen as follows. In all cases, we have set the rates 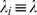. There is no evidence from biochemical studies that this is indeed the case. Rather, this choice of 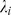 serves to reduce the number of free parameters and thus improve our intuition about the behavior of the various functions. As pointed out above, the non-exponential behavior of the decay patterns roots in different degradation rates 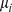. Therefore, in a first variant, all but the first absorption rates were the same, *i.e.* 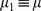 and 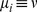 for all 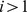. The corresponding absorption probability density has the form
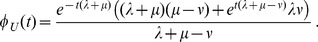
(9)


All red curves in Fig. 5 resulted in the smallest deviation to the experimental data by a fit of this equation together with (1) under the constraint 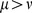. In this scenario, the equation is independent of the number of states *n* as long as 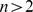.

In a second variant, we considered 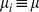 for all 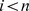 and 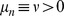. In this case, the lifetime probability density reads
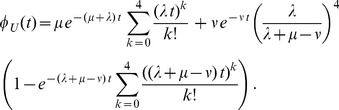
(10)


**Figure 8:**
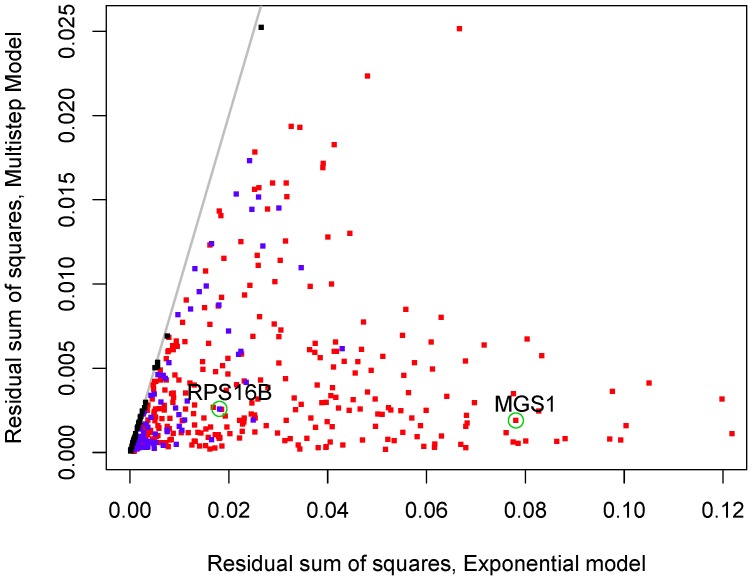
Comparison of fitting errors. The plot shows the residual sum of squares (*RSS*) after fitting the exponential model (abscissa) and the multistep model (ordinate) to the experiment data from Ref. [23]. Clearly, the multistep model leads to a considerable improvement of the fitting procedure, resulting in an average error reduction by almost one order of magnitude. Moreover, we also display the errors corresponding to the different categories in Fig. 5 as black, blue and red dots, respectively. The latter two represent the non-exponential patterns and typically imply a strong reduction of the fitting error. Additionally, we have highlighted the two representative cases *RPS16B* and *MGS1* belonging to the two non-exponential categories, as given in Fig. 3A. One may notice that there are some black dots, corresponding to the exponential decay patterns in Fig. 5. For these decay patterns, the fitting with a multi-step model does not provide a significant improvement of the fit compared to the exponential function.

All blue curves in Fig. 5 resulted in the smallest deviation to the experimental data by fitting this equation together with (1) under the constraint 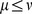. The improvement obtained by fitting the data with a multi-step model is displayed in Fig. 8. When one applies only the exponential model, the fitting error is typically large and less than half of the mRNAs lead to a residual sum of squares smaller than 0.01. In contrast, with a multi-step model more than 95% of the data can be fitted with great accuracy (*i.e.* RSS 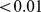). Nevertheless, some mRNA decay patterns are truly exponential. We have indeed rejected a more complex model, compared to the exponential function, if the more complex model did not improve the RSS by at least 10%. The black dots in Fig. 8 correspond to those decay patterns for which the exponential fit performed already very well and no significant improvement could be obtained with a multi-step fitting. A table with a list of all genes considered in this study and the optimal fit parameters to describe their decay is provided in the supporting information.

For the two exemplary decay patterns shown in Fig. 3A, all unknown rates were obtained from a fit to the experimental data of the mRNA level of *MGS1* and *RPS16B*. The lifetime probability densities given by Eqs. (9), for *MGS1*, or (10), for *RPS16B*, are then used in the fitting procedure via Eq. (1). For *MGS1*, we obtain the rates 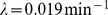, 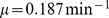 and 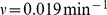, whereas for *RPS16B*, we have 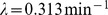, 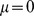 and 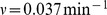. After determining these rates, we can now compute quantities that are not easily accessible experimentally, such as the lifetime probability distribution, the stationary age distribution at steady state expression and the age-dependent degradation rate.

### mRNA Lifetime Distribution, Patterns of Decay and Aging

We consider a cell culture under homogeneous and balanced growth conditions, in which the number of cells is kept constant by the balance between population growth and dilution. Under these ideal conditions, the number of mRNA molecules in the entire population for any given expressed gene fluctuates according to a Poisson distribution [43]. If *U* is the random lifetime of the mRNA molecules of a given gene and 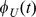 is its probability density, then
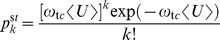
(11)is the stationary probability distribution for the number *k* of mRNA molecules and 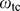 is the average transcription rate per cell. We will assume that 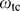 is a constant and that fluctuations due to transcriptional bursts in the different cells are averaged out at the population level. Eq. (11) holds when the creation and degradation of mRNA are in balance and is valid for any functional form of the lifetime distribution 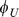 [27], [44].

To understand the fluctuations of the mRNA number at any time interval 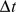 after the stop of transcription, we have to extend the theoretical description of Refs. [27], [44]. Let 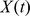 be the total number of mRNAs transcribed with a constant rate 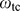 and 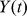 be the number of mRNAs still alive at any time *t*. The probability that *k* mRNAs are alive at time *t* is given by
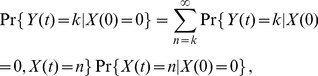
(12)where the second term on the right hand side arises from the process of mRNA generation, whereas the first term can be written as [27], [44]



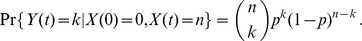
(13)Here, *p* denotes the probability that a single mRNA is still alive at time *t* under the condition that the number of mRNA transcribed until time *t* is *n*. The explicit form of *p* can be computed by exploiting a property of the Poisson processes, namely that conditional on 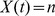 the origination times of the *n* events are uniformly distributed in the interval 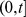 [27]. In the case of interest here, the origination time is in the interval 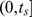 but we measure the number of mRNAs at time 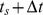. Thus, each mRNA has random origin *O* in the interval 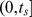 and random lifetime *U* given by Eq. (2). An mRNA has not been degraded until time 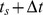 if 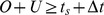 and thus
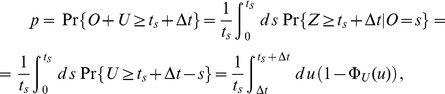
(14)where, in the last step we have used Eq. (2) and a suitable transformation of variables. Inserting Eq. (14) into Eq. (13), we can finally compute Eq. (12). If we assume that in decay experiments the cells were growing sufficiently long to have reached a stationary mRNA distribution such as in Eq. (11), we can consider the limit case 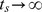 and the time-dependent distribution at a time delay 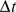 following the interruption of transcription reads
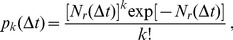
(15)where
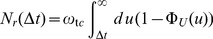
(16)is the average number of mRNAs after time 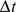 and 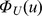 is the cumulative probability function defined in Eq. (2). Eq. (16) is particularly interesting for the present study because it gives the evolution of the average number of mRNAs after the interruption of transcription.

The time-dependent distribution (15) to find *k* mRNA molecules at time 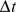 after the interruption of transcription is similar to the distribution obtained in Ref. [44] for a process after the start of transcription. The similarity, however, is restricted to the fact that both of them are Poisson distributions. The nature of the parameter 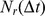 as in Eq. (16) is qualitatively different from the parameter found in Ref. [44].

The relative number of mRNAs after a time delay 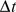 following the interruption of transcription is given by
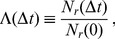
(17)which can be expressed explicitly as in Eq. (1). Hence, the half-life 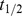 can be computed by solving 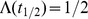. Fig. 3 shows both the experimental and the theoretical curves for the decay of 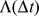 using the solutions (9) and (10) for the red and blue data, respectively. An analysis of the first and second derivative of 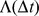 shows also that
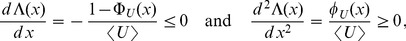
(18)which implies that the decay pattern is always decreasing and convex. Given the model independent nature of this relation, any bona-fide data must satisfy these criteria.

The residual lifetime *R* of a randomly chosen mRNA is also a random variable, whose distribution depends on the time 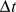 after the interruption of transcription. A detailed analysis presented in [44] provides a general form of the probability density of *R* for any value of 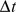


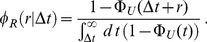
(19)


A simple integration of this quantity shows that if 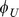 is exponential then 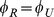. This is trivial because of the memoryless property of the exponential distribution. Nevertheless, for any other functional form of 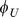 we find that 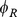 is a non-trivial function. The average residual lifetime is determined by the integral
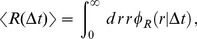
(20)for any 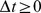. This expression enters into the determination of the residual protein synthesis capacity 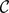 defined in Eq. (7). If the mRNAs are produced by a short transcriptional pulse, their residual lifetime probability density is not given by Eq. (19). Since in this case all mRNAs have the same age, the residual lifetime is given by



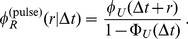
(21)In the following, we provide a heuristic derivation of Eq. (4). The transition from intact to degraded mRNA is given by the time-dependent rate 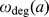. The exact definition of 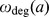 is given by means of the transition probability
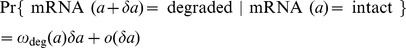
(22)for 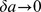. This transition probability means that given an intact mRNA of age *a*, its probability to be degraded within the next infinitesimal interval of time 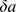 is 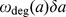. The probability 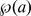 that the mRNA is still intact at age *a* is then defined as



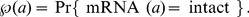
(23)Thus, using the conditional probabilities defined in (22) we obtain the following differential equation
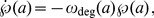
(24)whose solution with the initial condition 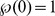 is given by



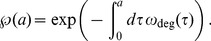
(25)Since, by the definition of 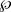 given in (23) it holds that 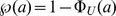, the differential equation (24) delivers the expression of 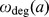 in terms of the probability density and its cumulative function as given in Eq. (4).

## Supporting Information

Table S1Summary and the details of our fitting results for all genes studied in this work. The data is organized as follows: Column 1: Gene id. Column 2: Category (0 = exponential = black, 1 = slow-fast = blue, 2 = fast-slow = red).Column 3: Parameter 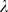 (transition rate) [1/min].Column 4: Parameter 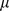, first degradation rate [1/min].Column 5: Parameter 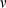, second degradation rate [1/min].Column 6: Residual sum of squares (RSS).Column 7: Mean lifetime 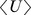 [min].Column 8: Half-life 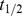 [min].Column 9–17: Experimental mRNA levels at time points 0,5,10,15,20,30,40,50,60 min after interruption of transcription (data from [23])(CSV)
